# Selenite Induces Cell Cycle Arrest and Apoptosis *via* Reactive Oxygen Species-Dependent Inhibition of the AKT/mTOR Pathway in Thyroid Cancer

**DOI:** 10.3389/fonc.2021.668424

**Published:** 2021-05-21

**Authors:** Zhen Cheng, Shuang Yu, Weiman He, Jie Li, Tianyi Xu, Junyu Xue, Peijie Shi, Shuwei Chen, Yanbing Li, Shubin Hong, Haipeng Xiao

**Affiliations:** ^1^ Department of Endocrinology, The First Affiliated Hospital of Sun Yat-sen University, Guangzhou, China; ^2^ Department of Breast and Thyroid Surgery, The First Affiliated Hospital of Sun Yat-sen University, Guangzhou, China; ^3^ Zhongshan School of Medicine, Sun Yat-sen University, Guangzhou, China; ^4^ Department of Head and Neck Surgery, Sun Yat-sen University Cancer Center, Guangzhou, China

**Keywords:** thyroid cancer (TC), sodium selenite, the AKT/mTOR pathway, cell cycle, apoptosis

## Abstract

Thyroid cancer is the most common endocrine malignancy, and its incidence has increased in the past decades. Selenium has been shown to have therapeutic effects against several tumors. However, its role in thyroid cancer and its underlying molecular mechanism remains to be explored. In the present study, we demonstrated that sodium selenite significantly decreased cell viability and induced G0/G1 cell cycle arrest and apoptosis in thyroid cancer cells in a dose-dependent manner. Transcriptomics revealed that sodium selenite induced intracellular reactive oxygen species (ROS) by promoting oxidative phosphorylation. Increased intracellular ROS levels inhibited the AKT/mTOR signaling pathway and upregulated EIF4EBP3. Intracellular ROS inhibition by N-acetylcysteine (NAC) ameliorated the cellular effects of sodium selenite. The *in vitro* findings were reproduced in xenograft thyroid tumor models. Our data demonstrated that sodium selenite exhibits strong anticancer effects against thyroid cancer cells, which involved ROS-mediated inhibition of the AKT/mTOR pathway. This suggests that sodium selenite may serve as a therapeutic option for advanced thyroid cancer.

## Introduction

The increase in the worldwide incidence of thyroid cancer over the past decade has been one of the fastest among cancers (1). According to the latest data, thyroid cancer is the most common malignancy of the endocrine system and is the ninth most common malignancy in the world (2). Differentiated thyroid cancer accounts for the majority of thyroid cancers, and approximately 10% of these cases cannot be cured using conventional treatments and can develop into metastatic disease (3). Anaplastic thyroid cancer is the most aggressive subtype of thyroid cancer, with a median overall survival of <10 months (4). Therefore, novel treatment strategies are urgently needed for the management of advanced thyroid cancer patients.

The cell cycle is a highly conserved cellular process consisting of four consecutive stages: G1, S, G2, and M. The normal progression of the cell cycle is regulated by various cyclin-dependent kinases (CDKs) and their partner cyclins. Continuous cell proliferation caused by uncontrolled cell division is one of the most important pathological manifestations involved in cancer progression. Among the complex cell cycle regulatory mechanisms, reactive oxygen species (ROS) have been shown to have an important impact on DNA synthesis, DNA stability, and cell cycle progression (5, 6). It is well established that cancer cells exhibit consistently high levels of ROS due to genetic and metabolic changes (7). The continuing ROS production forces cancer cells to develop effective ROS detoxification mechanisms, and the dependence on the antioxidant system increases cancer cells’ vulnerability to oxidative stress (8). By increasing oxidant production above the toxicity threshold, tumor cells can be killed *via* cell cycle arrest and apoptosis, while normal cells would be preserved. Thus, strategies aimed at inhibiting abnormal cell proliferation by altering the redox state in tumor cells open new avenues in cancer treatment (9).

Selenium (Se) is an essential trace mineral for humans. Its dysregulation has been linked to cancer and other complex diseases (10). Studies have shown that malignant cells are more sensitive to selenium than normal cells (11, 12). Importantly, selenium supplementation has been shown to be inversely associated with cancer risk and it improved the efficacy of anticancer drugs and the overall clinical outcomes of cancer patients (13). As the most commonly used selenium compound, sodium selenite (SS) has been reported to inhibit the growth of liver, breast, and peritoneal cancers by regulating redox homeostasis (14–16). Interestingly, decreased serum selenium concentration was associated with an increased incidence of thyroid cancer (17), and a significantly decreased selenium concentration was found in thyroid cancer tissues (18, 19). Herein, we tested the hypothesis that SS may have an anti-thyroid cancer effect by performing a series of *in vitro* and *in vivo* experiments.

## Materials and Methods

### Cell Lines

The thyroid cancer cell lines KTC-1, BCPAP, 8505C, and KHM-5M cells were obtained from the Cell Culture Collection of the Chinese Academy of Sciences (Shanghai, China). The sources of the TPC-1 (a thyroid cancer cell line) and Nthy-ori 3-1 cells (an immortalized thyroid epithelial cell line) were as previously described (20). The KTC-1, BCPAP, and KHM-5M cells were cultured in Roswell Park Memorial Institute (RPMI)-1640 (Gibco) with 10% fetal bovine serum (FBS; Gibco). The 8505C, TPC-1, and Nthy-ori 3-1 cells were cultured in Dulbecco’s Modified Eagle’s Medium (DMEM; Gibco) with 10% FBS. The cells were routinely cultured at 37°C in an atmosphere containing 5% CO_2_.

### Drugs

SS was purchased from Sigma (Cat#: S9133). It was dissolved in sterile water in a biosafety cabinet to a concentration of 50 mg/mL for storage at -20°C. A working solution was prepared using sterile water before use. N-acetylcysteine (NAC) was also purchased from Sigma (Cat#: A7250). The NAC ampoule was fully dissolved in sterile water in a 37°C water bath (to accelerate the dissolution) and then stored at -20°C. The working concentration of NAC used for cell culture was 2 mM, and the pH was adjusted to 7.0 before use.

### Cell Viability Assay

Cells were seeded in 96-well flat-bottom plates at a density of 1000 cells per well. After 24 h, the cells were exposed to different concentrations of SS with or without NAC. Cell viability was evaluated using Cell Counting Kit-8 (CCK8) assays (Dojindo Laboratories). After incubation with a medium containing CCK8 solution for 2 h, the absorbance was assessed using a Multimode Microplate Reader (Thermo Fisher Scientific). Half maximal inhibitory concentration (IC50) values were calculated using GraphPad 5.0.

### Colony Formation Assay

Cells were seeded in 6-well plates and treated with different concentrations of SS for 2 weeks. Colonies were then fixed with 4% paraformaldehyde for 15 min and stained with 5% crystal violet for 15 min. Photos of the colonies were taken after washing with double-distilled water.

### Cell Cycle Analysis

Cells were seeded in 6-well plates and treated with different concentrations of SS for 24 h. The cells were then harvested and fixed in 70% ethanol at -20°C overnight. Thereafter, they were incubated with RNase A and stained with propidium iodide (PI) at 37°C for 1 h. The cells were evaluated using a flow cytometer (Thermo Fisher Scientific). The distribution of cells according to the cell cycle phases was then analyzed using FlowJo 10.6.2.

### Apoptosis Analysis

Cells were seeded in 6-well plates and treated with different concentrations of SS for 24 h. Cells in the medium and adherent cells were all collected and stained using an Annexin V- fluorescein isothiocyanate (FITC)/PI Apoptosis Detection Kit (AD10; Dojindo Laboratories) according to the manufacturer’s protocol. Apoptotic cells were then analyzed using flow cytometry.

### Detection of Caspase-3/7 Activity and Hoechst Staining

Cells were seeded in 96-well plates (black plates, clear bottom with lid) and treated with different concentrations of SS for 24 h. The cells were then stained with Caspase-3/7 Green ReadyProbes Reagent (R37111; Thermo Fisher Scientific) and Hoechst-33342 (Beyotime Institute of Biotechnology) for 30 min, and the fluorescent signals were detected using FITC and 4’,6-diamidino-2-phenylindole (DAPI) filters.

### Western Blotting

Cells were harvested and lysed using lysis buffer for 15 min at 4°C. The protein concentration was assessed using a bicinchoninic acid (BCA) assay. Western blotting was carried out as described previously (21). The primary antibodies were as follows: phospho-AKT S437 (4060T; Cell Signaling Technology), total AKT (4691T; Cell Signaling Technology), phospho-mTOR S2448 (ab109268; Abcam), total mTOR (ab32028; Abcam), phospho-ERK1/2 T202/Y204 (#4370; Cell Signaling Technology), total ERK1/2 (#4695; Cell Signaling Technology), and β-actin (ab8226; Abcam).

### Detection of ROS

Cells were seeded in 96-well clear-bottom black plates and treated with 10 µM SS or phosphate-buffered saline (PBS). For some cells, NAC (2 mM) was added to eliminate the cellular ROS. After 6 h of treatment, the cells were incubated for 1 h in a medium containing 10 µM dichlorofluorescin diacetate (DCFH-DA) at 37°C. The cells were then washed twice and re-suspended in a growth medium for detection. The fluorescent signals were detected by microscope.

### qRT-PCR

After treating thyroid cancer cells with SS or PBS (control), total RNA extraction, cDNA synthesis, and qRT-PCR were performed as described previously (21). The expression of β-actin was used as the reference. The sequences of the primers are presented in **Supplementary Table 1**.

### RNA-Seq

Total RNA was extracted using TRIzol reagent and purified using oligo(dT)-attached magnetic beads. The purified RNA was fragmented into small pieces, and the first-strand cDNA was generated using random priming, followed by second-strand cDNA synthesis. After end repair, the cDNA was amplified by PCR, and the products were validated using an Agilent Technologies instrument. The final library was generated after heat denaturation and circularization of the PCR products. Thereafter, single-end 50-base reads were generated on a BGIseq500 platform (BGI-Shenzhen, China). The sequencing data were filtered using SOAPnuke, stored in FASTQ format, and mapped to HISAT2 (v2.0.4). Expression levels were calculated using RSEM (v1.2.12). The heatmap was constructed using pheatmap (v1.0.8). Kyoto Encyclopedia of Genes and Genomes (KEGG) enrichment analysis was performed using Phyper. There were two biological replicates for each experiment. A q value ≤0.05 based on the Bonferroni correction was considered statistically significant.

### siRNA Transfection

EIF4EBP3-targeting siRNA and negative control siRNA were purchased from YouBio (Changsha, China). Thyroid cancer cells were seeded in 6-well plates, cultured for 24 h until they reached 40–50% confluence, and then transfected with EIF4EBP3-targeting siRNA or negative control siRNA using a Lipo3000 Transfection Kit (Invitrogen) according to the manufacturer’s instructions. After 48 h of transfection, the cells were harvested and used for further experiments. The sequence of the negative control siRNA was: 5’- UUCUCCGAACGUGUCACGUTT-3’. The sequence of the EIF4EBP3-targeting siRNA was: 5’- CGCACAAUUUGAAAUGGAC-3’.

### Animal Studies

Animal studies were carried out according to the Guidelines of the Animal Investigation Committee and approved by the Ethical Committee of The First Affiliated Hospital of Sun Yat-sen University. KHM-5M cells (5×10^6^) or BCPAP cells (5×10^6^) were injected subcutaneously into the right flank of 4-week-old BALB/c nude mice. The mice with xenografts were then given 2, 4, or 8 mg/kg/day SS (200 µL/day; Cat#: S5261; Sigma) or saline through oral administration. For the SS+NAC group, pH-corrected NAC (30 mM) was added to the drinking water. Bodyweight and tumor size (using digital calipers) were assessed every 3 days, and the tumor volumes were calculated according to the formula: length × width^2^ × 0.5. All mice were sacrificed at the end of treatment. Tumor tissues and major organs were collected for immunohistochemical and hematoxylin and eosin (HE) staining.

### Immunohistochemical and HE Staining

Tumor tissues and major organs were fixed with 4% paraformaldehyde, dehydrated, embedded in paraffin, and cut into 5-µm-thick sections. The tissue sections were dewaxed (by heating at 60°C for 2 h), washed in xylene, and then rehydrated using a graded ethanol series. After antigen retrieval using microwave heating and blocking with 20% goat serum, the tissue sections were incubated with primary antibody (anti-Ki67 antibody; ab15580; Abcam) overnight at 4°C. Subsequently, immunodetection was carried out using a 3,3’-diaminobenzidine (DAB) peroxidase substrate kit (K5007; Dako) according to the manufacturer’s protocol. For HE staining, the slides were stained with hematoxylin and eosin sequentially after dewaxing and rehydration.

### Terminal Deoxynucleotidyl Transferase dUTP Nick End Labeling (TUNEL) Assay

Tissue sections were dewaxed and rehydrated as mentioned above, and then incubated with proteinase K for 30 min at 37°C. After washing with PBS, the tissue sections were incubated with TUNEL reaction solution for 60 min at 37°C in the dark and then dyed with DAPI and fixed in fluorescent mounting media.

### Statistical Analysis

Student’s t-test and one-way ANOVA analysis were used to determine statistical significance. Statistical analysis was performed using GraphPad Prism 5.0 (GraphPad Software, Inc., La Jolla, CA, USA). A p-value of <0.05 was considered statistically significant.

## Results

### SS Inhibits the Viability of Thyroid Cancer Cells

**Figure 1:**
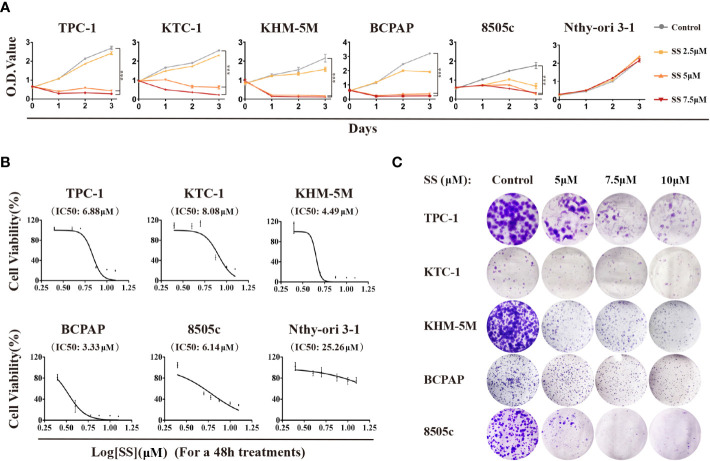
Cytotoxicity of SS in thyroid cancer cells. **(A)** Five thyroid cancer cell lines and one thyroid epithelial cell line were treated with different concentrations of SS for the indicated times. Cell viabilities were determined by CCK8 assays. **(B)** Half maximal inhibitory concentration (IC50) values were calculated using the Reed–Muench method. **(C)** Effect of SS on cell growth based on colony formation assays. SS, sodium selenite. ***P < 0.001.

To investigate the effect of SS on thyroid cancer, we first evaluated the cell viability by treating six thyroid cell lines with SS. As shown in **Figure 1A**, the viability of the five thyroid cancer cell lines treated with SS were all significantly reduced compared to the control group in a time- and dose-dependent manner. However, selenite had no significant inhibitory effect on the viability of the immortalized thyroid epithelial cell line Nthy-ori 3-1. The IC50 of selenite was higher in the thyroid cancer cells than in the Nthy-ori 3-1 cells (**Figure 1B**). The selenite dose-dependent inhibition of thyroid cancer cells was also demonstrated by the colony formation assays (**Figures 1C** and **S1A**). Notably, there was no significant difference in the anticancer effect of selenite between the BRAF wildtype (WT) tumor cells (TPC-1, KTC-1, and KHM-5M) and the BRAF^V600E^-positive tumor cells (BCPAP and 8505c). Taken together, these data showed that thyroid cancer cells were more sensitive to SS compared to human thyroid epithelial cells.

### SS Induces G0/G1 Cell Cycle Arrest and Apoptosis in Thyroid Cancer Cells

**Figure 2:**
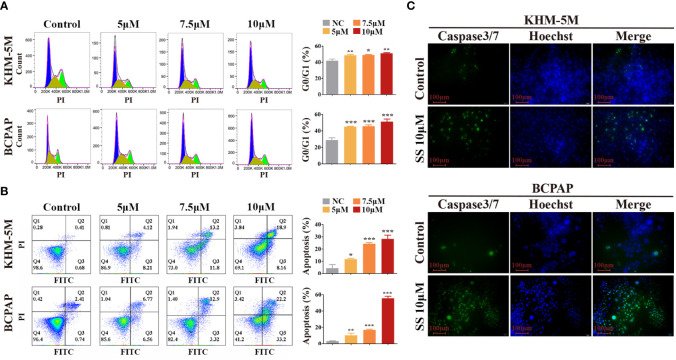
SS induces G0/G1 cell cycle arrest and apoptosis in thyroid cancer cells. **(A)** Cell cycle analysis was performed in KHM-5M and BCPAP cells after SS incubation for 24 h. **(B)** Apoptosis was evaluated by flow cytometry after treatment with the indicated concentrations of SS for 24 h. **(C)** Caspase-3/7 activity after SS treatment for 24 h. SS, sodium selenite. *P < 0.05; **P < 0.01; ***P < 0.001.

To investigate whether the modulation of the cell cycle contributes to the anti-proliferation effect of SS, we analyzed the cell cycle distribution in thyroid cancer cells by flow cytometry. Compared to the control group, the percentage of cells at the G0/G1 phase was increased in the SS group, while cells in other phases were decreased (**Figure 2A**). These results implied that SS effectively induced cell cycle arrest at the G0/G1 phase in a dose-dependent manner.

Typical morphological features of apoptosis were observed in thyroid cancer cells during prolonged selenite treatment (**Figure S1B**), suggesting that SS also induced apoptosis of thyroid cancer cells. Next, we performed Annexin V-FITC/PI staining assays followed by flow cytometry, and caspase activity detection, to confirm this. As shown in **Figures 2B, C**, the proportion of Annexin V-positive cells and the activity of caspase 3/7 were significantly increased after SS treatment. Moreover, the anti-apoptotic protein Bcl-2 was decreased after SS treatment (**Figure S1C**). Taken together, the results show that SS was capable of impeding cell cycle progression and triggering apoptosis in thyroid cancer cells.

### SS Induces ROS by Promoting the Oxidative Phosphorylation Pathway

**Figure 3:**
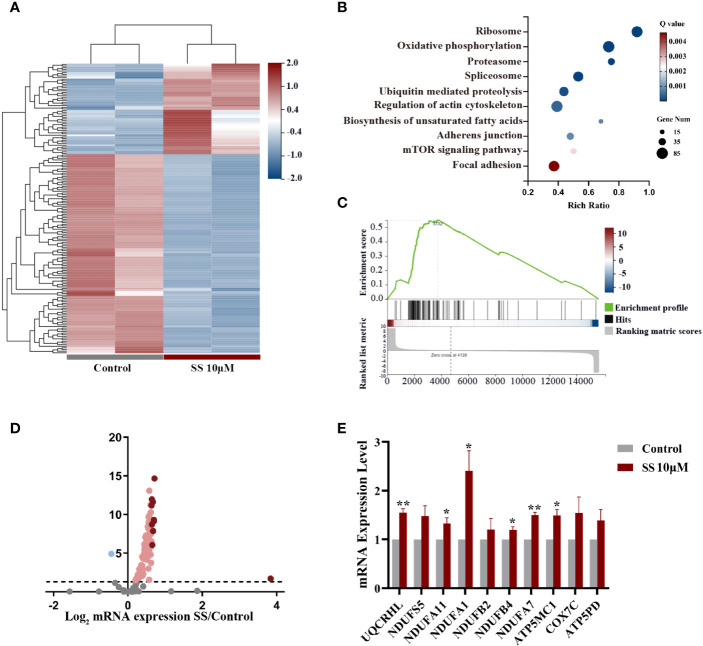
Gene expression analysis in thyroid cancer cells treated with SS. **(A)** Heatmap of differentially expressed genes between the SS and control groups. **(B)** Pathway enrichment analysis showing the top 10 changed pathways after SS treatment. **(C)** Single-sample gene set enrichment analysis (ssGSEA) was used to obtain the enrichment score of the oxidative phosphorylation pathway. **(D)** Volcano plot of genes enriched in the oxidative phosphorylation pathway (OXPHOS). **(E)** Relative RNA expression of the top 10 genes enriched in OXPHOS was validated by qRT-PCR in KHM-5M cells. SS, sodium selenite. *P < 0.05; **P < 0.01.

We next conducted a comprehensive RNA-seq analysis of early transcriptomic changes in KHM-5M cells to identify the underlying mechanism of SS. The principal component analysis clearly separated the SS-treated cells from the control cells. SS affected the expression of 4564 genes, of which 176 were altered by ≥2 fold (**Figure 3A**). We performed a KEGG pathway enrichment analysis of the differentially expressed genes to determine the critical pathways underlying the anticancer effect of SS. The top 10 enriched pathways, including the oxidative phosphorylation and mTOR signaling pathways, are shown in **Figures 3B, C**. Specifically, 105 genes were enriched in the oxidative phosphorylation pathway (**Figure 3D**). The expression of the top 10 enriched genes was validated by qRT-PCR in KHM-5M and three other thyroid cancer cells (**Figures 3E** and **S2A**). Among the genes enriched in the oxidative phosphorylation pathway, most encode components of the mitochondrial electron transport chain (mETC) complex, which is an important source of ROS. These results suggest that SS upregulated intracellular ROS levels by promoting mitochondrial oxidative phosphorylation.

### SS Inhibits Thyroid Cancer Cell Growth *via* ROS Induction

**Figure 4:**
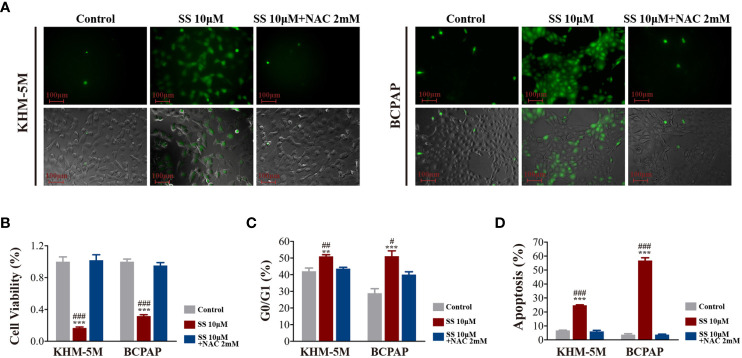
SS induced ROS in thyroid cancer cells. **(A)** Intracellular ROS levels detected by the ROS-sensitive fluorescent dye dichlorofluorescein diacetate (DCFH-DA) after incubation with SS or SS+NAC (2 mM) for 6 h. **(B)** Cell viability based on CCK8 assays, **(C)** cell cycle distribution based on flow cytometry, **(D)** apoptosis based on flow cytometry were detected after incubation with SS, PBS (control), or SS+NAC (2 mM) for 24 h. Data are expressed as mean ± SD. **P < 0.01; ***P < 0.001 *vs* control; ^#^P < 0.05; ^##^P <0.01; ^###^P < 0.001 *vs* SS+NAC group. SS, sodium selenite; NAC, N-acetylcysteine.

As the transcriptomic analysis suggested that oxidative phosphorylation is involved in the effects of SS, we detected the intracellular ROS levels in thyroid cancer cells using DCFH-DA. As shown in **Figure 4A**, SS treatment for 6 h significantly increased the cellular ROS levels, which were restored by the ROS inhibitor NAC (2 mM) (**Figure 4A**). Moreover, the SS-induced reduction in cell viability was restored by NAC (**Figure 4B**), while the SS-induced cell cycle arrest and apoptosis were also reversed by NAC (**Figures 4C, D**).

To confirm that the role of ROS regarding the effects of SS, we also performed RNA-seq in SS+NAC treated cells. There was distinct segregation between the control and SS groups but not between the control and SS+NAC groups (**Figure S2B**). The differentially expressed genes between the SS and SS+NAC groups were enriched in the oxidative phosphorylation pathway (**Figure S2C**). These results strongly indicated that ROS induction played a key role in mediating the effect of SS in thyroid cancer cells.

### AKT/mTOR/4E-BP3 Axis Contributes to the Anticancer Effect of SS

**Figure 5:**
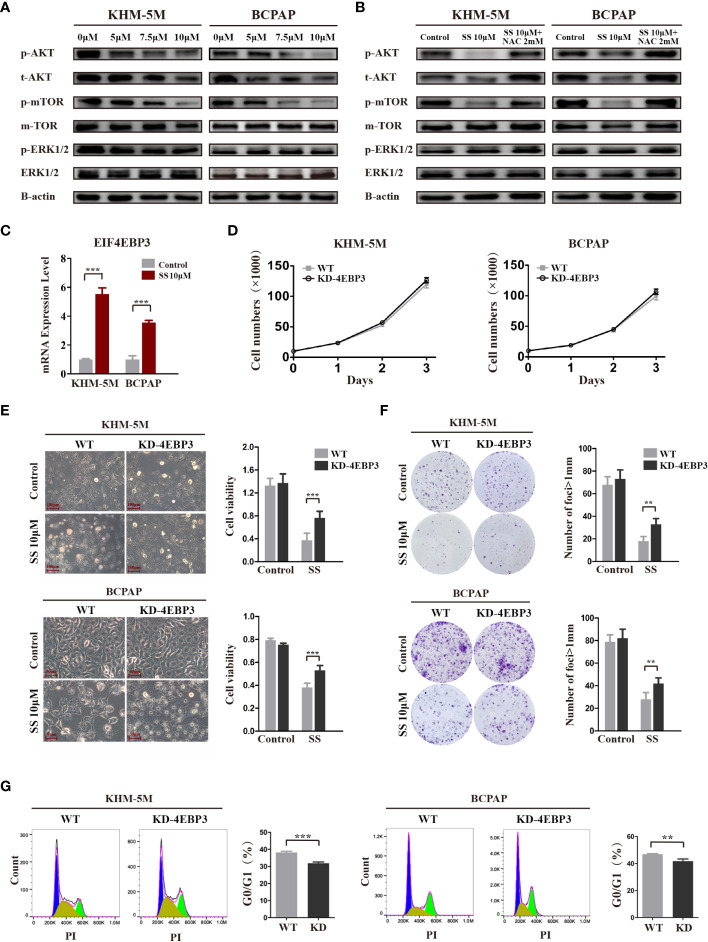
SS inhibits the AKT/mTOR/4E-BP3 axis in a ROS-dependent manner. **(A)** Thyroid cancer cells were treated with vehicle control or different concentrations of SS for 6 h, and the protein levels of p-AKT, total AKT, p-mTOR, total mTOR, p-ERK, and total ERK were detected by western blotting. The expression of β-actin was used as a reference. **(B)** NAC treatment reversed the inhibitory effect of SS on the AKT/mTOR pathway. **(C)** 4E-BP3 mRNA expression in SS treatment and control groups. **(D)** The proliferation of WT and 4E-BP3-knockdown cells under normal conditions. **(E)** Morphological changes, cell viability, and **(G)** percentage of G0/G1 phase cells in WT and 4E-BP3-knockdown groups after incubation with SS for 24 h. **(F)** Colony formation capacity of WT and 4E-BP3-knockdown cells after SS treatment. SS, sodium selenite; NAC, N-acetylcysteine. **P < 0.01; ***P < 0.001.

The AKT/mTOR and MAPK pathways are major pathways in the development of thyroid cancer. Therefore, we investigated the effect of SS on these two pathways in thyroid cancer cells. As shown in **Figure 5A**, SS treatment significantly decreased the levels of phosphorylated and total AKT in a dose-dependent manner, and the phosphorylation levels of mTOR were correspondingly reduced. In contrast, there were no changes in the protein levels of key molecules in the MAPK/ERK pathway, including phosphorylated and total ERK. Importantly, the inhibitory effect of SS on the AKT/mTOR pathway was reversed by NAC treatment (**Figure 5B**).

4E-BP3, encoded by the EIF4EBP3 gene, is an important molecule that mediates the effects of mTOR inhibitors (22). Our RNA-seq results showed that SS significantly upregulated EIF4EBP3 mRNA in KHM-5M cells, which was confirmed by qRT-PCR in KHM-5M and BCPAP cells (**Figure 5C**). Based on these results, we speculate that 4E-BP3 may be involved in the anticancer effects of SS. To confirm our hypothesis, we transiently knocked down 4E-BP3 using siRNA and assessed the cell behaviors. Although there was no significant difference in cell proliferation between WT and 4E-BP3-knockdown cells under normal conditions (**Figure 5D**), the latter cells had higher viability and clonogenicity after SS treatment (**Figures 5E, F**), and a significantly lower proportion of G0/G1 cells after SS treatment (**Figure 5G**). Taken together, our findings indicate that the effects of SS on thyroid cancer cells were mediated by the AKT/mTOR/4E-BP3 axis in a ROS-dependent manner.

### SS Inhibits the Growth of Thyroid Cancer *In Vivo*

**Figure 6:**
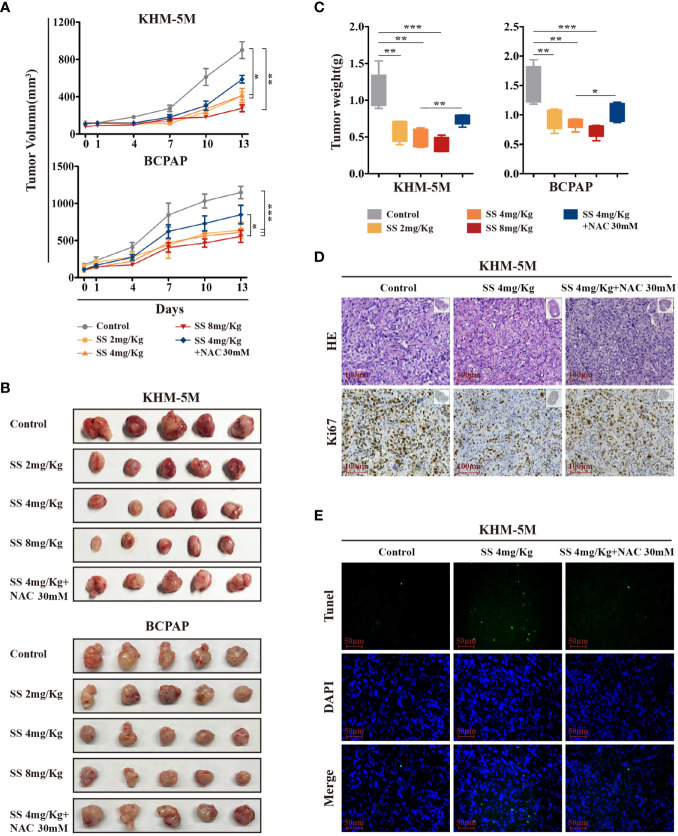
The therapeutic effect of SS in xenograft mouse models. **(A)** Tumor-bearing mice were treated with 0–8 mg/kg SS or SS+NAC for 14 days, and tumor volumes were measured using digital calipers. **(B)** Tumor tissues were removed and photographed after treatment. **(C)** Box-whisker plot of tumor weights. **(D)** Ki-67 staining and **(E)** TUNEL assay were used to evaluate the proliferation and apoptosis in dissected tumors from different groups. Data are expressed as mean ± SD. SS, sodium selenite; NAC, N-acetylcysteine. *P < 0.05; **P < 0.01; ***P < 0.001.

To investigate the anticancer effect of SS *in vivo*, thyroid cancer cells (KHM-5M or BCPAP) were injected into the right flank of BALB/c nude mice. Mice with xenografts were treated with 2, 4, or 8 mg/kg SS or saline (control) daily *via* oral administration. As shown in **Figure 6A**, xenograft tumors treated with SS exhibited slower growth than tumors in the control group, and the effect was dose-dependent. NAC treatment reduced the inhibitory effect of SS on the xenograft tumors (**Figures 6B, C**). Next, we evaluated the effect of SS on cell proliferation and apoptosis in the xenograft tumors using Ki67 staining and TUNEL assays. The results showed that the proportion of Ki67-positive cells in tumor tissues was significantly lower in the SS group than the control group, while the percentage of TUNEL-positive cells was significantly higher. The effects of SS on cell proliferation and apoptosis in xenograft tumors were reversed by NAC (**Figures 6D, E**). There were no significant differences in body weight between the experimental and control groups (**Figure S3A**). The toxicity of SS on the major organs of mice was evaluated by HE staining, with no obvious damage being observed between the SS and control groups (**Figures S3B, C**). These findings indicated that SS treatment inhibited thyroid cancer *via* ROS induction *in vivo*.

## Discussion

This is the first study to show that sodium selenite induces cell cycle arrest and apoptosis in thyroid cancer. By performing transcriptome sequencing, we revealed that sodium selenite stimulated the accumulation of intracellular ROS by upregulating mETC components and suppressing the AKT/mTOR pathway. An mTOR signaling repressor gene, EIF4EBP3, was found to be involved in the anticancer effect of selenite in thyroid cancer. Furthermore, the ROS inhibitor NAC abrogated the cytotoxicity of ROS and, thus, the anticancer effect of sodium selenite in thyroid cancer cells.

The cell cycle and apoptosis are two important determinants of the cell proliferation rate. The cell cycle checkpoint is a crucial mechanism to ensure high-fidelity cell division. Apoptosis is a form of programmed cell death, and the organelles and other proteins from apoptotic cells can be recycled by other cells. We showed that SS can effectively impede the cell cycle and induce apoptosis in thyroid cancer cells. Previous analyses of gene expression profiles in several cancer cells also showed that selenium specifically downregulated CYCLIN A, CYCLIN D, CDC25A, CDK4, and other cell cycle-related genes to induce cell cycle arrest (10, 23). Experiments in H22 hepatocarcinoma cells found that selenium nanoparticles exerted a more pronounced blocking effect on the G0/G1 phase than cisplatin (24). Therefore, the specific regulation of the tumor cell cycle and apoptosis by selenium makes it a potential tumor treatment option.

Among the diverse anticancer mechanisms of selenite (10, 25), the major one is its regulation of redox homeostasis, which is fundamental for maintaining cellular function and cell survival. Cancer cells are characterized by increased aerobic glycolysis, which is needed to fuel their uncontrolled growth (26). Because of the accumulation of ROS, upregulated antioxidant systems are vital for the survival of cancer cells (27), and these cells exhibit vulnerability to increased oxidative stress (7). Here we showed that SS treatment sharply decreased thyroid cancer cell viability by increasing ROS, which is consistent with previous findings. In prostate cancer cells, ROS production was indispensable for SS-induced decreased ATP levels and cell death (28). Treatment of liver cancer cells with pharmacological doses of SS under normoxic conditions caused intracellular oxidative stress while, under hypoxic conditions, the SS-induced accumulation of hydrogen selenide (H2Se) led to reducing stress (14). Through transcriptomic sequencing, we found that SS upregulated genes encoding the mitochondrial complex I and IV subunits in thyroid cancer cells. The mETC is a major source of intracellular ROS production (29), and the ubiquinone reduction site of complex I has the largest ROS production capacity among the four complexes in the mETC. Thus, the increased ROS caused by mitochondrial oxidative phosphorylation may be the main mechanism underlying the effect of SS against thyroid cancer.

ROS accumulation activates multiple pathways and affects cell survival. As a classic oncogenic pathway in the development of thyroid cancer, the AKT/mTOR pathway is also closely related to metabolic stress and cellular nutritional status (30, 31). Our study showed that increased ROS levels inhibited the AKT/mTOR pathway, which in turn led to apoptosis. Eliminating ROS using NAC reversed the inhibitory effect of SS on the AKT/mTOR pathway. Furthermore, as an important therapeutic target in various types of cancer, including thyroid cancer (32), mTOR exerts various impacts on multiple genes related to cell proliferation, among which 4E-BP3 is an important effector molecule. Here, we found a significant upregulation of EIF4EBP3 in cells treated with SS, and further functional experiments revealed that knocking down EIF4EBP3 mitigated the effect of SS in thyroid cancer. Consistent with our findings, a previous study reported that inhibition of mTOR by rapamycin upregulated EIF4EBP3 and induced cancer cell death (22).

Analyses of xenograft mice further validated the anticancer effect of SS on thyroid cancer, which was mitigated by NAC treatment. The reversal effect of NAC was not as powerful as in the *in vitro* experiments, which might be caused by its low bioavailability *in vivo*. While our study and studies in other cancer types have demonstrated the therapeutic effects of sodium selenite (13, 33, 34), its clinical feasibility remains to be evaluated in clinical trials.

In conclusion, this study provides evidence that sodium selenite effectively suppresses thyroid cancer growth. We also revealed a novel mechanism involving upregulation of mETC components, increased intracellular ROS, and inhibition of the AKT/mTOR pathway. The results provide a basis for the management of aggressive thyroid cancer by sodium selenite supplementation.

## Data Availability Statement

The datasets presented in this study can be found in online repositories. The names of the repository/repositories and accession number(s) can be found below: https://www.ncbi.nlm.nih.gov/geo/query/acc.cgi?acc=GSE168843).

## Ethics Statement

The animal study was reviewed and approved by the Ethical Committee of the First Affiliated Hospital of Sun Yat-sen University.

## Author Contributions

ZC, SH, and HX conceived and designed the study. ZC, SY, and WH performed the experiments and analyzed the data. TX, JX, and PS conducted data curation and analyzed the data. JL, SC, and YL were responsible for software analysis and data visualization. ZC wrote the first draft of the manuscript. SH and HX reviewed and edited the manuscript. All authors contributed to the article and approved the submitted version.

## Funding

This work was supported by the Youth Program of the National Natural Science Foundation of China (No. 81802677) and Guangzhou Science and Technology Project (No. 201803010057).

## Conflict of Interest

The authors declare that the research was conducted in the absence of any commercial or financial relationships that could be construed as a potential conflict of interest.
