# Supplementary material for: Selenite Induces Cell Cycle Arrest and Apoptosis via Reactive Oxygen Species-Dependent Inhibition of the AKT/mTOR Pathway in Thyroid Cancer
Source: Front Oncol. 2021 May 21;11:668424. doi: 10.3389/fonc.2021.668424 (PMC8176115; doi:10.3389/fonc.2021.668424)
Supplement: Supplementary file 1 [file DataSheet_1.pdf]

Supplementary Material

Supplementary Table 1. Primers information

RT-PCR	
h-UQCRHL-F	GACTGGAGGACGAGCAAAAG
h-UQCRHL-R	TCTCTCACTGTTGTTAGGGG
h-NDUFS5-F	TGCACATGGAATCGGTTATACTC
h-NDUFS5-R	CCGAAGCAAACACTCTACGAAAT
h-NDUFA11-F	GCCGAAGGTTTTTCGTCAGTA
h-NDUFA11-R	GGAGGATTGAGTGTGACTCTGT
h-NDUFA1-F	ATGTGGTTCGAGATTCTCCCC
h-NDUFA1-R	CCTGTGGATGTACGCAGTAGC
h-NDUFB2-F	GGAGGCCGCCTTTTCAGAA
h-NDUFB2-R	GGAAGGATCAGGATACGGAAAGT
h-NDUFB4-F	ATGTCGTTCCCAAAGTATAAGCC
h-NDUFB4-R	GAAGCAGGTACTCTCGTTTCAG
h-NDUFA7-F	TGCAGCTACGCTACCAGGA
h-NDUFA7-R	GAGCTTGTGGCTAGGACCC
h-ATP5MC1-F	CTGTTGTACCAGGGGTCTAATCA
h-ATP5MC1-R	GTGGGAAGTTGCTGTAGGAAG
h-COX7C-F	GTGTCTTACTACAAGGAAGGGTG
h-COX7C-R	GGTCCGTAGGAGCCACTATGA
h-ATP5PD-F	GCTGGGCGAAAACTTGCTCTA
h-ATP5PD-R	CCAGTCGATAGCTGGTGGATT
h-EIF4EBP3-F	CGGTTACTTTGCTGCTCCTC
h-EIF4EBP3-R	GTGCGTCATCGGGTATCTCT
h-β-ACTIN -F	CATCACCATTGGCAATGAGC
h-β-ACTIN -R	GCCATGCCAATCTCATCTTG

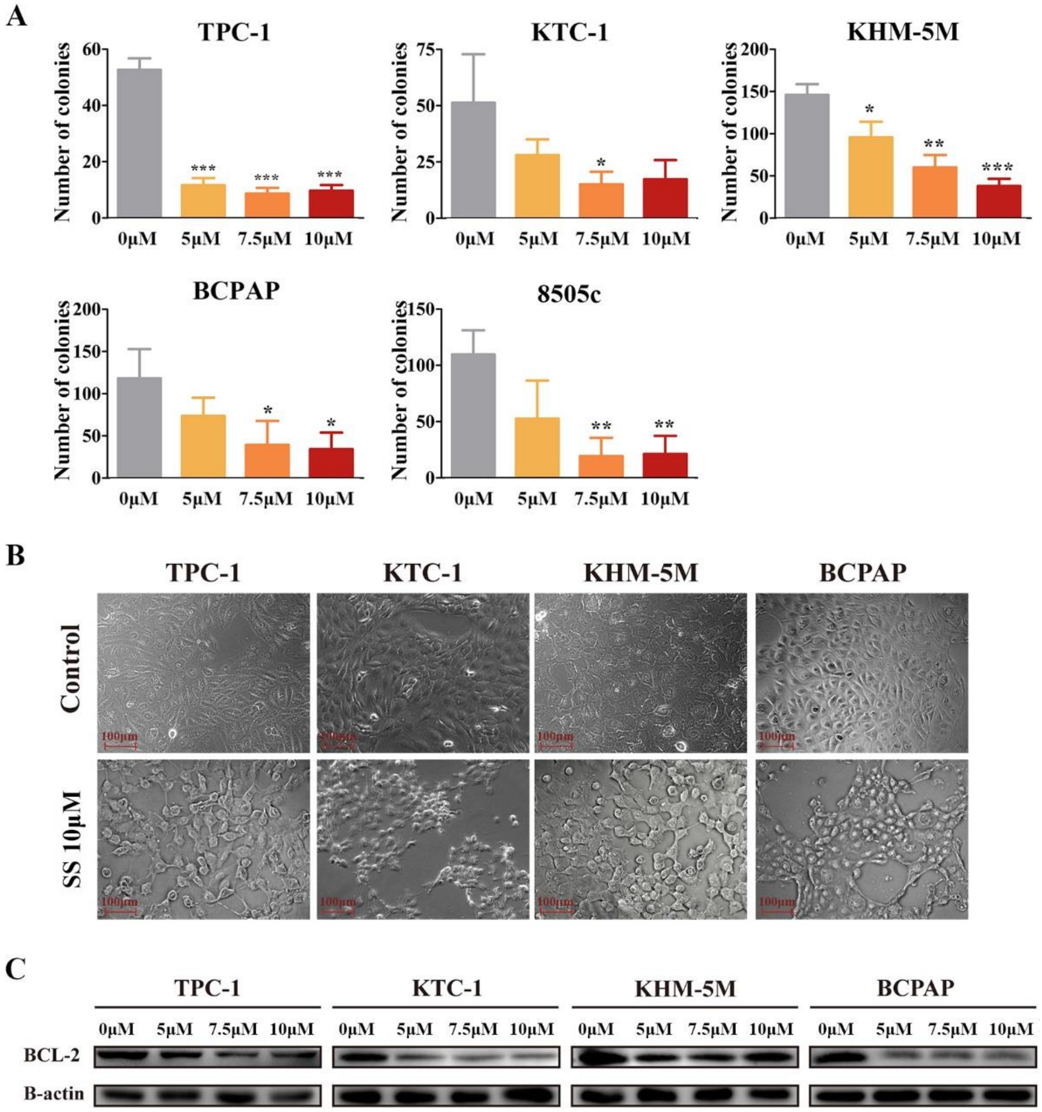

Supplementary Figure 1. SS induces thyroid cancer cell apoptosis. (**A**) The number of colonies in control and SS treatment groups. (**B**) Morphological changes of cancer cells were observed under microscope and (**C**) the protein levels of Bcl-2 were detected after treated with indicated concentration of selenite for 24 h. *P<0.05; **P<0.01; ***P<0.001. SS: Sodium selenite.

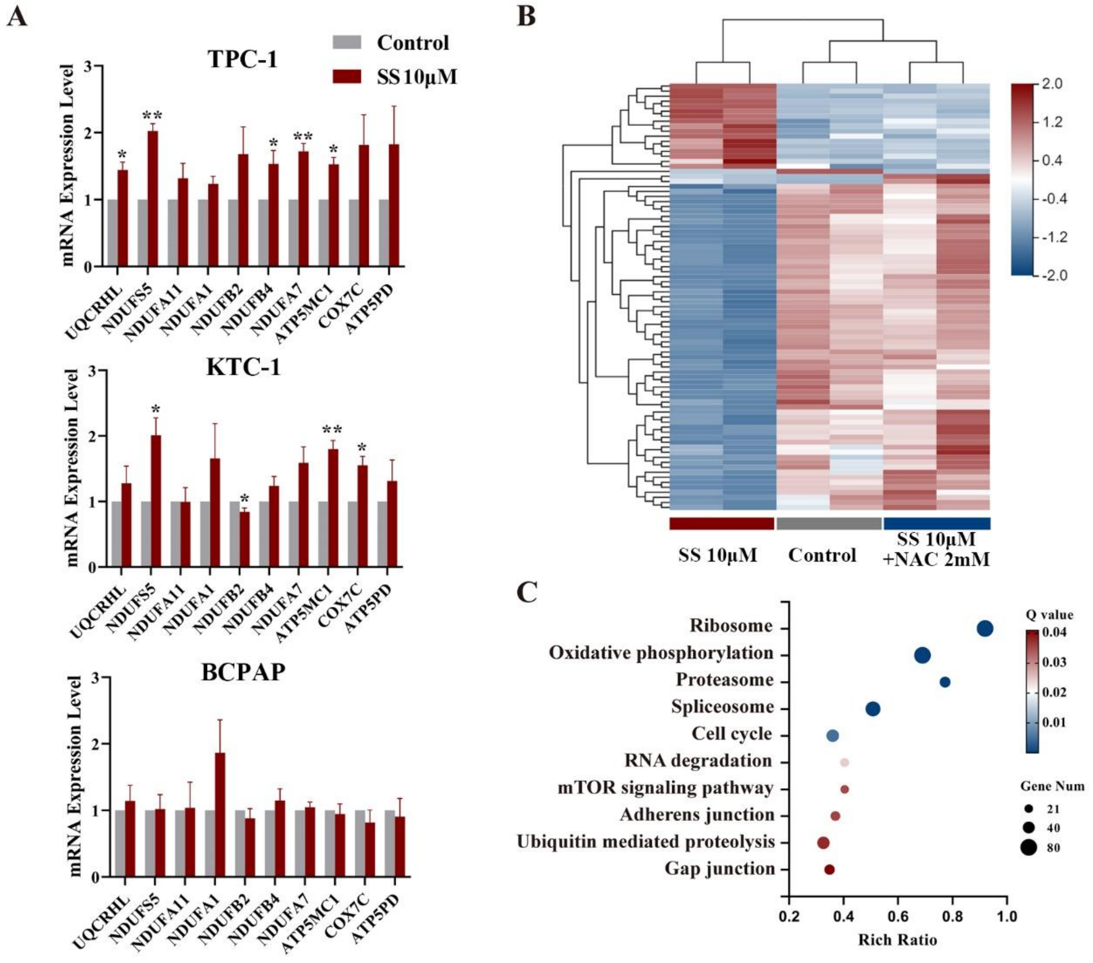

Supplementary Figure 2. Transcriptomic changes induced by SS. (**A**) Relative RNA expressions of top 10 genes enriched in oxidation phosphorylation pathway were validated by qRT-PCR in TPC-1, KTC-1 and BCPAP. (**B**) Hierarchical cluster analysis of differentially expressed genes in SS, control, and SS+NAC group. (**C**) Pathway enrichment analysis of differentially expressed genes between SS group and SS+NAC group. *P<0.05; **P<0.01. SS: Sodium selenite. NAC: N-acetylcysteine.

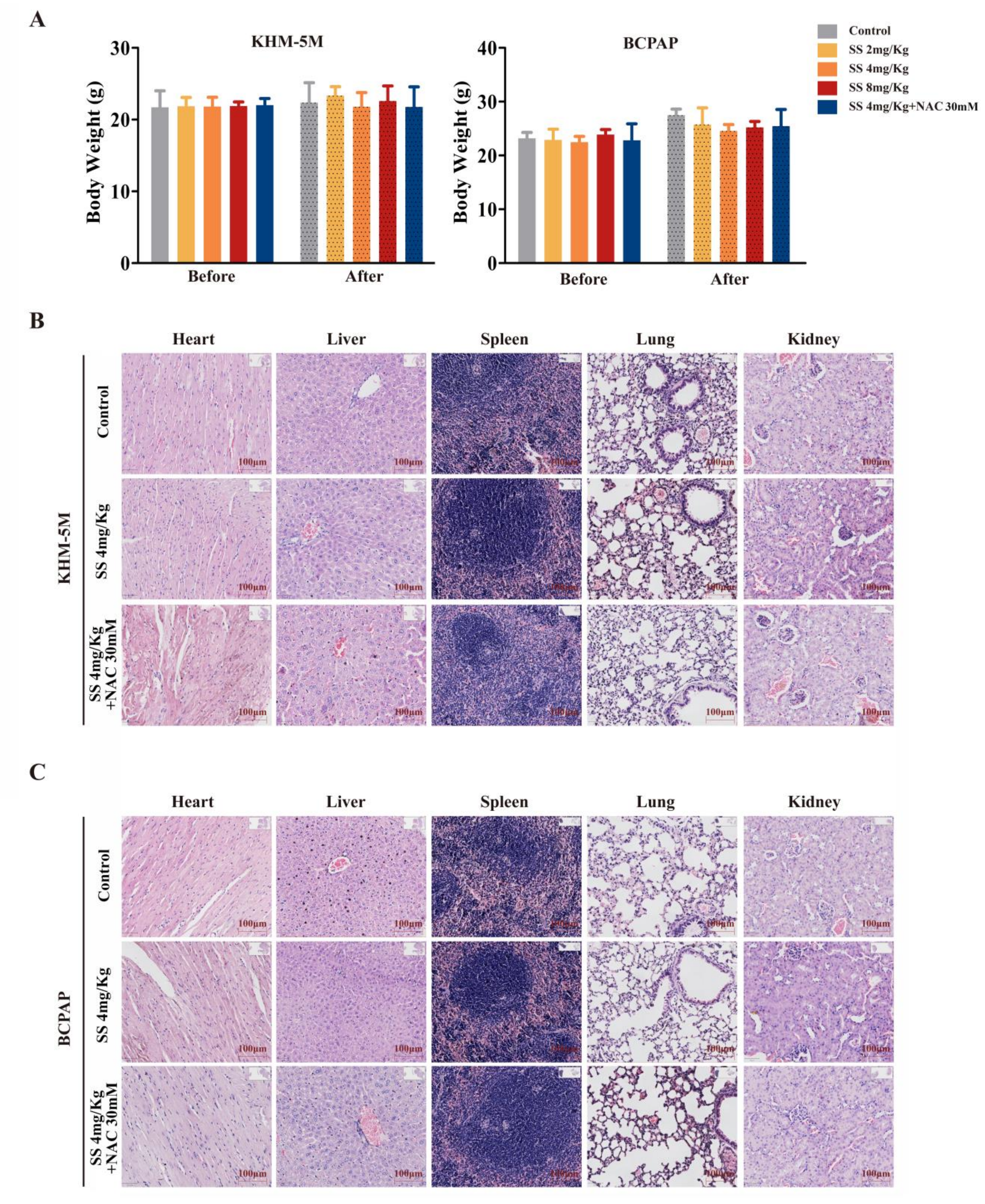

Supplementary Figure 3. Animal safety of sodium selenite treatment. (**A**) The body weights of mice in different groups before and after treatment. (**B**) HE staining of major organs of KHM-5M or (**C**) BCPAP xenografts mice after experiments. SS: Sodium selenite. NAC: N-acetylcysteine.

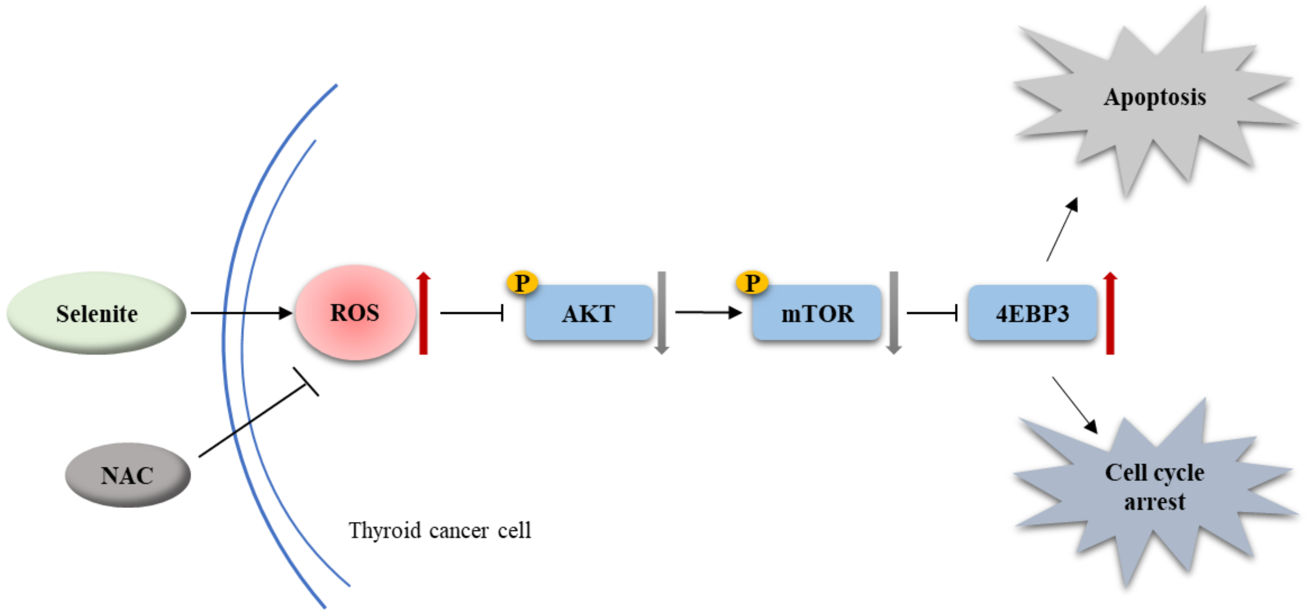

Supplementary Figure 4. Schematic picture to clarify the anticancer function of selenite in thyroid cancer cell.
